# Classification of lung nodules in CT scans using three-dimensional deep convolutional neural networks with a checkpoint ensemble method

**DOI:** 10.1186/s12880-018-0286-0

**Published:** 2018-12-03

**Authors:** Hwejin Jung, Bumsoo Kim, Inyeop Lee, Junhyun Lee, Jaewoo Kang

**Affiliations:** 1 Department of Computer Science and Engineering, Korea University, Seoul, Republic of Korea; 2 Interdisciplinary Graduate Program in Bioinformatics, Korea University, Seoul, Republic of Korea

**Keywords:** Convolutional neural network, Deep learning, Ensemble, Lung nodule, Lung cancer

## Abstract

**Background:**

Accurately detecting and examining lung nodules early is key in diagnosing lung cancers and thus one of the best ways to prevent lung cancer deaths. Radiologists spend countless hours detecting small spherical-shaped nodules in computed tomography (CT) images. In addition, even after detecting nodule candidates, a considerable amount of effort and time is required for them to determine whether they are real nodules. The aim of this paper is to introduce a high performance nodule classification method that uses three dimensional deep convolutional neural networks (DCNNs) and an ensemble method to distinguish nodules between non-nodules.

**Methods:**

In this paper, we use a three dimensional deep convolutional neural network (3D DCNN) with shortcut connections and a 3D DCNN with dense connections for lung nodule classification. The shortcut connections and dense connections successfully alleviate the gradient vanishing problem by allowing the gradient to pass quickly and directly. Connections help deep structured networks to obtain general as well as distinctive features of lung nodules. Moreover, we increased the dimension of DCNNs from two to three to capture 3D features. Compared with shallow 3D CNNs used in previous studies, deep 3D CNNs more effectively capture the features of spherical-shaped nodules. In addition, we use an alternative ensemble method called the checkpoint ensemble method to boost performance.

**Results:**

The performance of our nodule classification method is compared with that of the state-of-the-art methods which were used in the LUng Nodule Analysis 2016 Challenge. Our method achieves higher competition performance metric (CPM) scores than the state-of-the-art methods using deep learning. In the experimental setup ESB-ALL, the 3D DCNN with shortcut connections and the 3D DCNN with dense connections using the checkpoint ensemble method achieved the highest CPM score of 0.910.

**Conclusion:**

The result demonstrates that our method of using a 3D DCNN with shortcut connections, a 3D DCNN with dense connections, and the checkpoint ensemble method is effective for capturing 3D features of nodules and distinguishing nodules between non-nodules.

## Background

Lung cancer accounts for more than a quarter of all cancer deaths and is one of the major threats to human health in both men and women worldwide [1]. For these reasons, early detection and examination of lung nodules, which might be malignant, is necessary [2]. Radiologists spend countless hours carefully detecting small spherical-shaped nodules in computed tomography (CT) images. Moreover, a considerable amount of effort and time is required for radiologists to determine whether detected nodules are malignant. Therefore, a reliable computer aided detection (CAD) system is needed to assist radiologists. High performance CAD systems can be utilized as a decision support tool for radiologists and reduce the cost of manual screenings [3–5].

In general, computer aided detection and diagnosis systems for lung cancer perform the following three tasks: delineation of lungs, nodule candidate detection, and false positive reduction. Nodule candidate detection in delineated lungs is limited by a high false positive rate [6]. The high number of false positive nodules makes CAD difficult to be employed for clinical use. It is essential to reduce the number of false positive nodules as much as possible to move on to the stage of precise nodule assessment [7, 8]. For these reasons, we focus on solving the false positive reduction task.

**Fig. 1:**
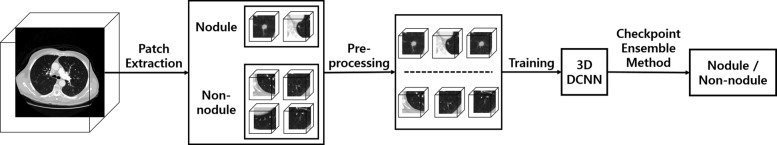
Pipeline of our nodule classification method. Three dimensional patches of nodules and non-nodules are extracted and pre-processing is conducted to balance the ratio of nodules to non-nodules. A three dimensional deep convolutional neural network (3D DCNN) with shortcut layer connections and a 3D DCNN with dense layer connections are trained on the prepared dataset for nodule classification. Finally, the checkpoint ensemble method is applied to boost performance of our nodule classification method

Our method uses three dimensional deep CNNs (3D DCNNs) that have novel layer connections (shortcut and dense) and a much deeper structure than the shallow networks commonly used in existing research studies. We increase the dimension of DCNN from 2 to 3 to effectively capture the spherical features of lung nodules. In addition, we apply a checkpoint ensemble method to boost nodule classification performance. While we employ the widely used layer connections to build a deep structured CNN, increasing the dimension of CNN from 2 to 3 and the checkpoint ensemble method help improve performance. Figure 1 shows the pipeline of our nodule classification method. We extract three dimensional patches of nodule candidates and non-nodule candidates. Pre-processing is conducted to balance the number of nodule candidates and non-nodule candidates. After pre-processing, our 3D DCNNs are trained on the prepared dataset.

The remainder of this paper is organized as follows. We first introduce the related works on the nodule classification task. The details of our 3D DCNN and the checkpoint ensemble method are described in the “Method” section. The dataset, pre-processing step, experimental setups, and experimental results are reported in the “Experiment and result” section. The discussion and final conclusions are provided in the “Conclusion” section.

As the performance of medical imaging devices improves, the number of high quality medical images continues to increase. The rapid increase in the number of medical images is already a burden to medical experts. The need for efficient diagnostic decision support tools that provide consistent results, reliable performance, and rapid processing has emerged [3, 5]. Several studies on effective medical image analysis methodology have been conducted. Medical image analysis methods have evolved from pattern recognition using a simple image filter and machine learning methods based on feature engineering to deep learning based methods. Deep learning methods that automatically extract features from images have become the most popular approach. Deep learning is applied to various types of medical images such as lung CT scans [9], mammograms [10], histopathology images [11], and PET/CT images [12], and achieves state-of-the-art analysis performance.

Several studies in the field of lung CT scan analysis have devoted their efforts to developing robust and efficient lung nodule classification methods. Since using shape features of lung nodules was the dominant method, most studies focused on designing representative hand-crafted features of lung nodules. Unfortunately, the wide variation in lung nodules in CT scans prevents conventional machine learning models with hand-crafted features from performing consistently [13, 14].

As deep learning models produced promising results for image classification, deep learning nodule classification methods that did not use manual features were proposed to overcome the problems of conventional machine learning methods that used hand-crafted features. A convolutional auto-encoder that was employed to automatically capture the shapes of nodules outperformed traditional machine learning models with hand-crafted features [15, 16]. Also, nodule classification methods using simple 2D convolutional neural networks (2D CNNs) trained on cross-sectional images were proposed [17, 18]. These methods outperformed the methods that use a neural network or a stacked auto-encoder (SAE).

Although the methods using 2D CNN enhanced performance, they could not utilize all the 3D information of CT scans, which is the most important feature of CT scans. Several studies applied 2D CNN with some adjustments to address this problem. To capture 3D information, various cross-sectional images presented in various views were used [9, 19, 20]. Specifically, three CNNs trained on three different-sized images in axial, sagittal, and coronal views, respectively, were used. The last layers of the CNNs were put together to predict the final result [19]. Another method used additional hand-crafted 3D features. Pre-defined 3D features of nodules were manually extracted and features of 2D nodules were extracted using a 2D DCNN. Both sets of features were combined and used as input to a Random Forest (RF) classifier [21].

To overcome the limitations of the methods that use 2D CNN, which cannot solve the fundamental problem, methods using 3D CNN have recently been proposed. A method using a shallow 3D CNN that can receive a 3D patch as an input was proposed [22]. The authors used three 3D CNNs with different input sizes. The three 3D CNNs were trained separately and the final class prediction was made by the linear combination of their results [23]. Furthermore, entire pipelines that can perform nodule detection and false positive reduction were introduced. A specialized object detection deep learning model was employed to find lung nodule candidates from 2D CT slices. Also, a 2D CNN [9] and a 3D CNN [24] were applied to classify nodules for reducing false positives.

All the above-mentioned methods achieved high performance, but there is still room for improvement. As nodule classification is a complex task due to the numerous and diverse features of nodules, a deep network structure is needed. In this paper, we propose a nodule classification method that uses an extremely deep three dimensional convolutional neural network, which vastly differs from a shallow 3D CNN commonly used in existing nodule classification studies. In addition, an ensemble method is used to help boost nodule classification performance.

## Method

### Layer connection

When training deep convolutional neural networks (DCNNs), the weights of DCNNs are updated by calculating the gradient of the loss function. The gradient is initially calculated in the last layer and flows toward the first layer by sequentially updating itself. The gradient at a layer depends on the gradient of its previous layer. This updating process is called back-propagation [25]. Also, the depth of the network is important in back-propagation. While back-propagation works well in shallow networks, gradients gradually vanish as they move from the last layer to the first layer of an extremely deep structured CNN. This is known as the vanishing gradient problem which is mainly attributed to poor back-propagation, and makes the training process less efficient [26, 27]. Therefore, neatly stacking convolution layers in DCNN does not guarantee high performance.

While several approaches such as normalized initialization [27–30] and batch normalization [31] have been proposed to address this notorious problem, one of the most effective approaches involves connecting layers to allow gradients pass more quickly and directly. Shortcut connections and dense connections are two representative layer connection types. They successfully alleviate the gradient vanishing problem and help deep structured CNNs obtain low and high level features of objects.

**Fig. 2:**
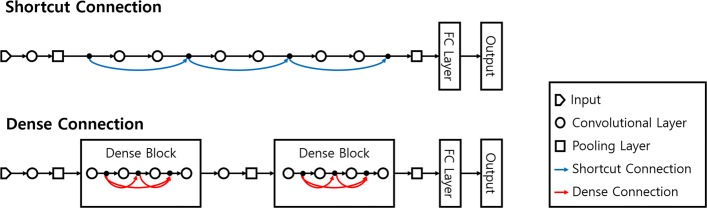
Two different types of layer connections: shortcut connection and dense connection. The top diagram illustrates CNN with shortcut connections and the bottom diagram illustrates CNN with dense connections

Shortcut connections and dense connections are used for connecting the previous layer to the next layer to ensure efficient gradient propagation. The shortcut connections are indicated by blue curved lines in Fig. 2. When the gradient passes through deeply stacked CNNs without shortcut or dense connections, it gradually vanishes. However, connections allow gradient to skip one or more convolutional layers [32], and directly pass backwards without vanishing. The top diagram of Fig. 2 shows the simple structure of CNN with the shortcut connections. The layers of CNN with shortcut connections are stacked in the same way they are in CNN without connections.

In the bottom diagram of Fig. 2, the dense connections which are indicated by red curved lines connect each layer to every other layer. The main difference between a shortcut connection and dense connection is density. Dense connections are another representative convolutional layer connection type and an extremely dense version of shortcut connections [33]. Convolutional layers are connected by dense connections and a series of connected layers forms a dense block. These blocks are repeatedly stacked to construct a DCNN. The bottom diagram of Fig. 2 shows the simple structure of CNN with dense connections.

### Model description

**Fig. 3:**
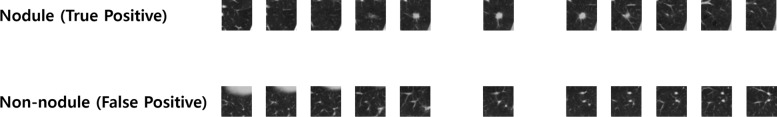
Sample patches of nodules. The top row of patches and the bottom row of patches show consecutive patches of a true positive nodule and a false positive nodule, respectively. All the patches are displayed in an axial view

To solve the nodule classification problem, we use two deep convolutional neural networks with shortcut connections and dense connections, respectively. Shortcut connections and dense connections, which are similar but distinct, make it possible for DCNNs to be trained successfully by overcoming the vanishing gradient problem. In addition, to address 2D DCNN’s inability to consider the spherical shape of nodules, we modified the 2D DCNN structure. Figure 3 shows some consecutive patches of true positive nodules and false positive nodules. These patches are displayed in an axial view. The patches located in the middle of the figure are generally used as input for nodule classification methods based on 2D CNN. However, it is difficult to distinguish nodules from non-nodules based on only the fragmented sections. To address this, nodule classification methods based on 2D CNN have used additional three dimensional features [17–21]. Also, examining consecutive sections together can be helpful in distinguishing nodules.

For more effective 3D feature extraction, we modified the dimension of DCNN from 2 to 3, instead of manually creating 3D features using feature engineering. To construct our 3D DCNNs, we increased the dimension of all the components of DCNN (convolutional and pooling layers) from 2 to 3. The architectures of our 3D shortcut connection DCNN and 3D dense connection DCNN are shown in Tables 1 and 2, respectively. Each network is constructed by stacking a number of connected convolutional layers or dense blocks, instead of simply stacking individual convolutional layers one after the other. The depth of our 3D DCNNs is the same as that in the original study of shortcut connection and dense connection [32, 33]. The output size of the last layer is set to 2 for classifying lung nodules (nodule or non-nodule). The 3D dense connection DCNN is much deeper and wider than the 3D shortcut connection DCNN. To demonstrate the importance of input size, we construct 3D DCNNs with different input sizes. The input sizes of 64 ×64 ×64 and 48 ×48 ×48 are used for the 3D dense connection DCNN and the 3D shortcut connection DCNN, respectively.

**Table 1:** The structure of the 3D shortcut connection DCNN

Layer name	Structure
convolution_1	7×7×7 conv3×3×3 max pool
convolution_2	$\begin {bmatrix} 3 \times 3 \times 3 \text { conv} \\ 3 \times 3 \times 3 \text { conv} \end {bmatrix}$ ×2
convolution_3	$\begin {bmatrix} 3 \times 3 \times 3 \text { conv} \\ 3 \times 3 \times 3 \text { conv} \end {bmatrix}$ ×2
convolution_4	$\begin {bmatrix} 3 \times 3 \times 3 \text { conv} \\ 3 \times 3 \times 3 \text { conv} \end {bmatrix}$ ×2
convolution_5	$\begin {bmatrix} 3 \times 3 \times 3 \text { conv} \\ 3 \times 3 \times 3 \text { conv} \end {bmatrix}$ ×2
	7×7×7 avg pool1000-d FCsoftmax

**Table 2:** The structure of the 3D dense connection DCNN

Layer name	Structure
	7×7×7 conv
	3×3×3 max pool
Dense block	$\begin {bmatrix} 1 \times 1 \times 1 \text { conv}\\ 3 \times 3 \times 3 \text { conv} \end {bmatrix}$ ×6
Transition	1×1×1 conv2×2×2 avg pool
Dense block	$\begin {bmatrix} 1 \times 1 \times 1 \text { conv} \\ 3 \times 3 \times 3 \text { conv} \end {bmatrix}$ ×12
Transition	1×1×1 conv2×2×2 avg pool
Dense block	$\begin {bmatrix} 1 \times 1 \times 1 \text { conv} \\ 3 \times 3 \times 3 \text { conv} \end {bmatrix}$ ×24
Transition	1×1×1 conv2×2×2 avg pool
Dense block	$\begin {bmatrix} 1 \times 1 \times 1 \text { conv} \\ 3 \times 3 \times 3 \text { conv} \end {bmatrix}$ ×16
	7×7×7 avg pool1000-d FCsoftmax

We conduct model training and testing using a single machine with the following configuration: Intel(R) Core(TM) i7-6700 3.30GHz CPU with NVIDIA GeForce GTX 1070 Ti 8GB GPU and 48GB RAM. The Adam optimizer [34] and the cross entropy loss function are used for training our models. The learning rate starts from 0.001 and is divided by 2 after every 3 epochs. The code for our 3D shortcut connection DCNN and 3D dense connection DCNN is available at the GitHub repository (https://github.com/hwejin23/LUNA2016).

### Ensemble

**Fig. 4:**
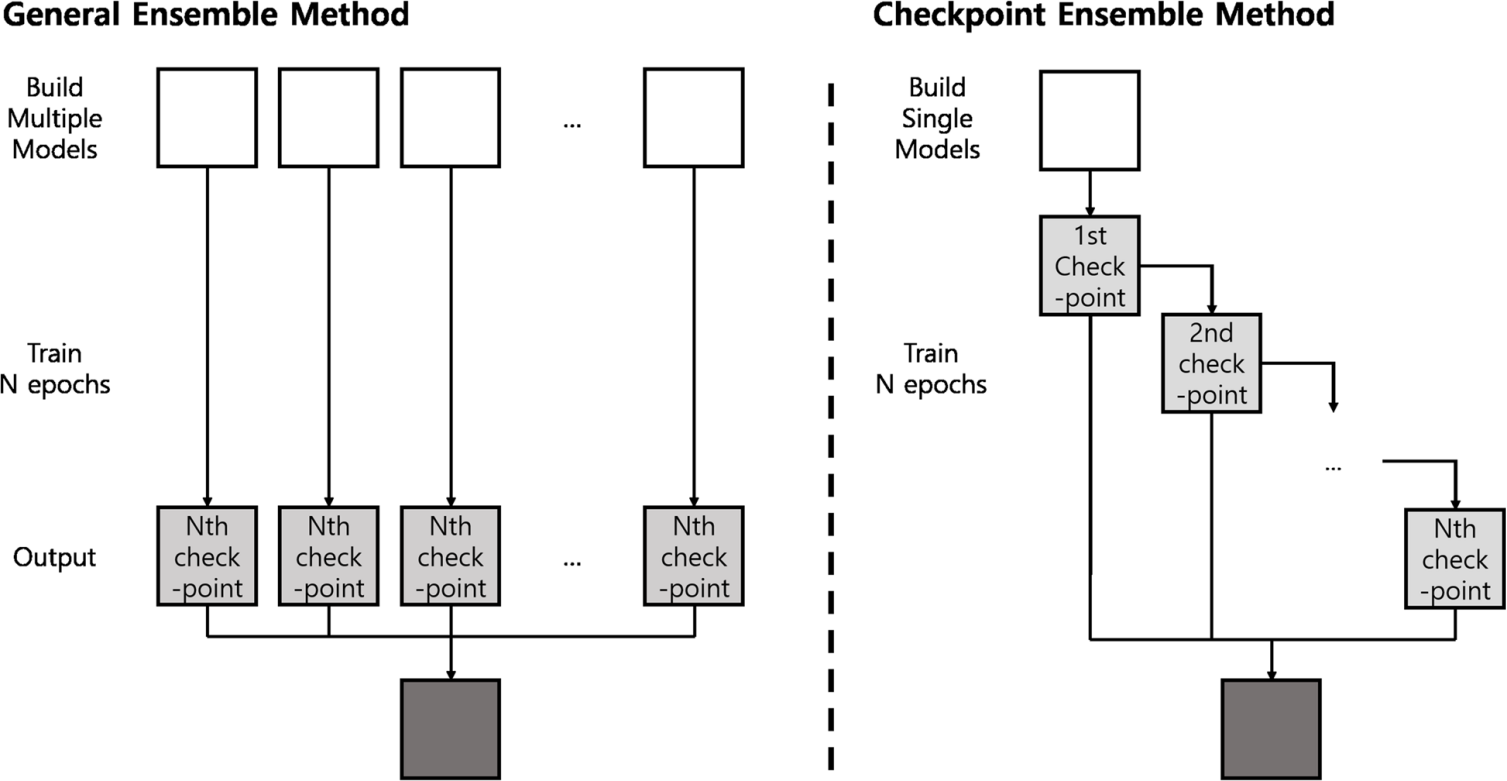
Two different types of ensemble methods. The general ensemble method (left) and checkpoint ensemble method (right)

We use an ensemble method that aggregates the results of multiple trained models to boost performance. In general, increasing the number of ensemble members and varying the structures of models enhance ensemble performance by decreasing the variance of prediction [35]. The left diagram of Fig. 4 illustrates the general ensemble method. When adopting the general ensemble method, a number of randomly initialized identical models are sufficiently trained and model weights are stored at the end of training. Among the stored weights from different models, the model weights that contribute the most to improving performance are used as ensemble members. The results of ensemble members are aggregated by averaging the results or majority vote.

Numerous samples must be used for the lung nodule classification task. The number of parameters increases when the number of layers and dimension of DCNN increase. Training DCNNs many times to obtain several ensemble members is extremely time consuming; thus, applying the general ensemble method which requires a sufficient number of ensemble members is impractical. Therefore, instead of the general ensemble method, we use the checkpoint ensemble method [36–38]. In the checkpoint ensemble method, no additional training for several randomly initialized identical models is needed. In other words, a randomly initialized model is trained only once. The checkpoint ensemble method uses model weights (checkpoints) which are stored in the middle of the training phase as shown in the right diagram of Fig. 4.

Since LUNA16 consists of 10 subsets, we train our DCNN on 9 subsets in turn and test it on the remaining subset. We define an epoch as the point where the DCNN completes training on all 9 subsets. In the training phase, the model weights are stored at the end of every epoch. Since non-nodules are randomly down-sampled and nodules are augmented for the training set, which is explained in more detail in the “Pre-processing” section, the composition of the training set is different for each epoch. Thus, the model is trained on a different set at every epoch, and not on the same set.

Due to their deep network structure, training our 3D DCNNs on three dimensional input images and a great amount of training data for one epoch using our machine takes around one day. Due to a limited amount of time, we use six ensemble members for each of the following DCNNs with different input sizes: 3D shortcut connection DCNN with input size 48, 3D shortcut connection DCNN with input size 64, 3D dense connection DCNN with input size 48, and 3D dense connection DCNN with input size 64. The results of the ensemble members are aggregated by averaging the confidence scores. In addition, to determine whether the ensemble method is effective for various types of DCNNs, the ensemble method is applied to each DCNN.

## Experiment and result

### Dataset

We used the public dataset from the LUng Nodule Analysis 2016 (LUNA16) challenge [39] (https://luna16.grand-challenge.org/). According to the challenge organizers, they selected 888 CT scans out of a total of 1018 CT scans from the publicly available reference database of the Lung Image Database Consortium and Image Database Resource Initiative (LIDC-IDRI) [40]. Identified nodules were extracted using the following nodule detection algorithms: ISICAD, SubsolidCAD, and LargeCAD [41–43]. The candidate nodules were manually annotated by four experienced thoracic radiologists. Each radiologist classified the nodules as nodules ≥3 mm, nodules <3 mm, or non-nodules [44, 45]. The challenge organizers used a total of 1186 nodules deemed to be larger than 3 mm by three or four radiologists as the true positive findings. The remaining nodules were considered as false positive findings. There are 1557 true positive and 753,418 false positive samples in the dataset. For 10-fold cross-validation, the challenge organizers divided the LUNA16 dataset into 10 subsets. Though the challenge ended on January 3, 2018, the dataset and the evaluation script are still available online.

### Pre-processing

The dataset provided by the organizers of LUNA16 has about 460 times more non-nodules than nodules. While using an abundant number of training samples can help train the model, training on an imbalanced dataset can lead model to be over-fitted [46]; hence, we apply several sampling and augmentation methods to address the data skewness problem. We repeatedly sample non-nodules and nodules for every epoch. We decided to include all the nodules in the training set. However, non-nodules are randomly down-sampled until there are 100 times more non-nodules than nodules in the training set. In other words, the training set for every epoch contains all the nodules and 100 times more randomly sampled non-nodules than nodules. The training set is further balanced by up-sampling the nodules, applying the following augmentation methods. Each sample image is slightly shifted to a random position. The random center shifting method prevents all objects from being located in the center of the patch. In addition, each sample is randomly rotated by 90 degrees using three orthogonal axes (X, Y, and Z). These augmentation methods balance the training set. Pre-processing is conducted on all 10 subsets and our models are trained on a sufficient number of nodule samples for every epoch.

### Evaluation metric

In the LUNA16 challenge, performance was evaluated using Free Response Receiver Operating Characteristic (FROC) and Competition Performance Metric (CPM). Sensitivity and the average number of false positives per scan are used for generating the FROC curves. Sensitivity is defined as Eq (1) where TP is true positives, FP is false positives, and FN is false negatives. In the FROC curves, sensitivity is plotted as a function of the average number of false positives per scan. The CPM score is defined as the average sensitivity at the following seven predefined false positive points: 0.125, 0.25, 0.5, 1, 2, 4, and 8. We also use a confusion matrix to show the true positive rate, false positive rate, true negative rate, and false negative rate for better performance comparison.

1 $$ Sensitivity = \frac{TP}{TP + FN}   $$

### Result

All of our experimental setups are listed in Table 3. S48 and S64 denote the experimental setups which use the 3D shortcut connection DCNN without the ensemble method. Similarly, D48 and D64 denote the experimental setups which use the 3D dense connection DCNN without the ensemble method. 48 and 64 refer to the input size of the DCNNs. ESB-S48 and ESB-S64 denote the experimental setups which use the 3D shortcut connection DCNN with the checkpoint ensemble method, and ESB-D48 and ESB-D64 denote the experimental setups which use the 3D dense connection DCNN with the checkpoint ensemble method. The following setups use six checkpoints respectively: ESB-S48, ESB-S64, ESB-D48, and ESB-D64. ESB-S denotes the experimental setup in which both the 3D shortcut DCNN with an input size of 48 and the 3D shortcut DCNN with an input size of 64 are used. ESB-D denotes the experimental setup in which both the 3D dense DCNN with the input size of 48 and the 3D dense DCNN with the input size of 64 are used. Both ESB-S and ESB-D use the checkpoint ensemble method. ESB-BEST denotes the setup using the ensemble method with the best checkpoints which are obtained for each type of DCNN. Finally, ESB-ALL denotes the experimental setup that uses the checkpoint ensemble method with all the checkpoints of all the DCNN types.

**Table 3:** Experimental setups

Setup name	Model type	Input size	# of checkpoints	Ensemble
S48	3D shortcut DCNN	48	1	X
S64	3D shortcut DCNN	64	1	X
D48	3D dense DCNN	48	1	X
D64	3D dense DCNN	64	1	X
ESB-S48	3D shortcut DCNN	48	6	O
ESB-S64	3D shortcut DCNN	64	6	O
ESB-S	3D shortcut DCNN	48	6	O
		64	6	
ESB-D48	3D dense DCNN	48	6	O
ESB-D64	3D dense DCNN	64	6	O
ESB-D	3D dense DCNN	48	6	O
		64	6	
ESB-BEST	3D shortcut DCNN	48	1	O
		64	1	
	3D dense DCNN	48	1	
		64	1	
ESB-ALL	3D shortcut DCNN	48	6	O
		64	6	
	3D dense DCNN	48	6	
		64	6	

Table 4 provides performance comparison of our nodule classification method in each experimental setup. The performance in S64 is better than that in S48 and the performance in D64 is better than that in D48. Thus, the DCNNs using a large input size of 64×64×64 obtain better results than the DCNNs using a smaller input size of 48×48×48. Regardless of input size, the 3D shortcut connection DCNN achieves better performance than the 3D dense connection DCNN. This demonstrates that the 3D shortcut connection DCNN are more effective than the 3D dense connection DCNN. Moreover, applying the checkpoint ensemble method improves the overall performance of the 3D DCNNs. CPM scores of 0.899 and 0.885 are obtained in ESB-S and ESB-D, respectively, in which the checkpoint ensemble method is used regardless of the input size. These are the highest scores obtained by a DCNN. Applying the checkpoint ensemble method further improves the performance of DCNNs. ESB-BEST which uses the checkpoint ensemble method obtains the CPM score of 0.897. Finally, using all the checkpoints for the ensemble members (ESB-ALL) obtains the highest CPM score of 0.910. The performance comparison shows that using diverse ensemble members helps enhance nodule classification performance. The ensemble method reduces model variance and helps models make unbiased predictions.

**Table 4:** Performance comparison of our nodule classification method in each experimental setup

	0.125	0.25	0.5	1	2	4	8	CPM
S48	0.691	0.788	0.851	0.891	0.910	0.934	0.945	0.859
S64	0.736	0.818	0.880	0.911	0.932	0.950	0.960	0.884
D48	0.676	0.765	0.839	0.894	0.922	0.938	0.953	0.855
D64	0.710	0.800	0.870	0.902	0.924	0.943	0.958	0.872
ESB-S48	0.655	0.739	0.863	0.927	0.962	0.973	0.976	0.871
ESB-S64	0.633	0.744	0.870	0.943	0.974	0.980	0.980	0.875
ESB-S	0.683	0.813	0.911	0.954	0.969	0.982	0.982	0.899
ESB-D48	0.645	0.736	0.816	0.908	0.954	0.975	0.980	0.859
ESB-D64	0.646	0.736	0.834	0.919	0.962	0.977	0.981	0.865
ESB-D	0.679	0.778	0.878	0.937	0.963	0.981	0.981	0.885
ESB-BEST	0.734	0.814	0.895	0.934	0.957	0.971	0.976	0.897
ESB-ALL	0.720	0.842	0.914	0.954	0.974	0.982	0.982	0.910

**Fig. 5:**
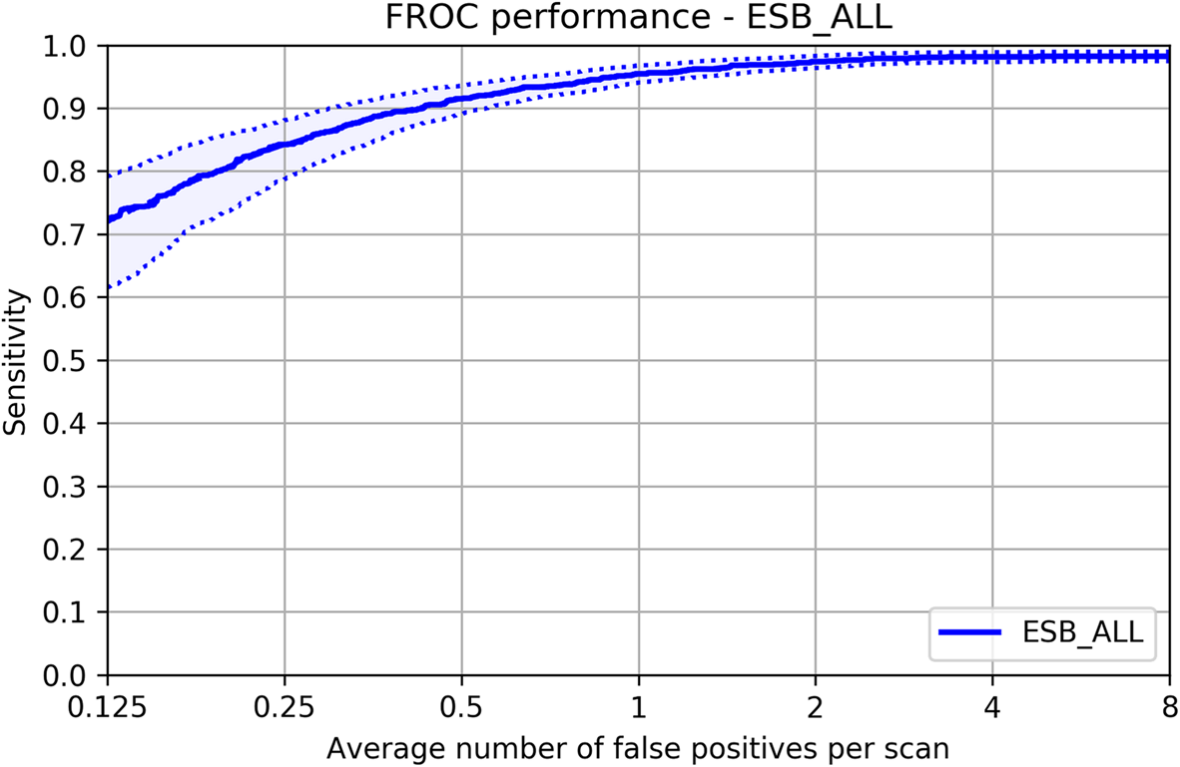
FROC curve of our method tested on LUNA16 dataset in the experimental setup ESB-All. The average number of false positives per scan ranges from 0.125 to 8

Tables 5 and 6 show the confusion matrices of D48 and ESB-ALL, respectively. Among all our experimental setups, the worst performance is obtained in setup D48, and the best performance is achieved in ESB-ALL. Even though the lowest CPM score is obtained in D48, a high true positive rate of 0.913 and a high true negative rate of 0.984 as well as a low false positive rate of 0.016 and a low false negative rate of 0.087 are also obtained in D48. Better results are obtained in ESB-ALL. Both the false positive rate of 0.007 and false negative rate of 0.067 decrease, and both the true positive rate of 0.933 and true negative rate of 0.993 increase. The best CPM score is obtained in ESB-ALL, as shown by the FROC curve presented in Fig 5. These results demonstrate that the nodule classification performance of our method is highly consistent.

**Table 5:** Confusion matrix of experimental setup D48 in which the worst performance is obtained

		Predicted class	
	D48	Nodule	Non-nodule
Actual	Nodule	0.913	0.087
Class	Non-nodule	0.016	0.984

**Table 6:** Confusion matrix of experimental setup ESB-ALL in which the best performance is obtained

		Predicted class	
	EBS-ALL	Nodule	Non-nodule
Actual	Nodule	0.933	0.067
Class	Non-nodule	0.007	0.993

The performance comparison of several existing nodule classification methods is provided in Table 7. Table 7 shows the results of our method in experimental setups D48 and ESB-ALL. The CPM lowest score of our method obtained in D48 is still higher than that of the other existing methods. Furthermore, our method obtained better performance than other methods in ESB-ALL. Sensitivity values at most false positives per scan points obtained in ESB-ALL are higher than those obtained in other setups. This shows that our nodule classification method can accurately classify nodules in various setups.

**Table 7:** Performance comparison of the state-of-the-art methods and our method

	Method	0.125	0.25	0.5	1	2	4	8	CPM
LUNA16CAD	2D CNN	0.113	0.165	0.265	0.465	0.596	0.695	0.785	0.440
LungNess	2D CNN	0.453	0.535	0.591	0.635	0.696	0.741	0.797	0.635
iitem03	2D CNN	0.394	0.491	0.570	0.660	0.732	0.795	0.851	0.642
[22]	3D CNN	0.517	0.602	0.720	0.788	0.822	0.839	0.856	0.735
LUNA16CAD	3D CNN	0.640	0.698	0.750	0.804	0.847	0.874	0.897	0.787
[9]	2D CNN	0.734	0.744	0.763	0.796	0.824	0.832	0.834	0.790
DIAG_CONVNET [23]	3D CNN	0.636	0.727	0.792	0.844	0.876	0.905	0.916	0.814
UACNN	2D CNN	0.655	0.745	0.807	0.849	0.880	0.907	0.925	0.824
CUMedVis [24]	3D CNN	0.677	0.737	0.815	0.848	0.879	0.907	0.922	0.827
D48	3D CNN	0.676	0.765	0.839	0.894	0.922	0.938	0.953	0.855
ESB-ALL	3D CNN	0.720	0.842	0.914	0.954	0.974	0.982	0.982	0.910

Compared with existing methods that use 2D CNN with a complex structure or 2D CNN with extra three dimensional features [9], our 3D DCNN method can effectively capture and extract 3D features of lung nodules without using additional features. Moreover, our method greatly outperforms the state-of-the-art methods using 3D CNN [22–24]. They use shallow 3D CNNs while our method uses 3D DCNNs. We show that three dimensional deep convolutional neural networks outperform shallow CNNs on the nodule classification task.

## Conclusion

In this paper, we used two 3D deep convolutional neural networks with shortcut connections and dense connections, respectively, for the nodule classification task. The 3D shortcut connection DCNN and the 3D dense connection DCNN were able to effectively obtain general as well as distinctive features of lung nodules, and alleviate the vanishing gradient problem. In addition, the three dimensional structure of DCNN is suitable for extracting spherical-shaped nodule features. We applied a checkpoint ensemble method to our 3D DCNNs to boost performance. The performance of our 3D DCNNs was measured on the LUNA16 dataset which is publicly available. Our nodule classification method significantly outperformed the state-of-the-art nodule classification methods. Though we used DCNNs with shortcut and dense connections, both of which are widely used, increasing the dimension of DCNNs from 2 to 3 and using the checkpoint ensemble method helped improve performance. For future work, we plan to develop an automatic lung nodule detection algorithm that can be used to find nodule candidates and apply it to our nodule classification method.

## Abbreviations

2D: 2 dimensional
3D: 3 dimensional
CAD: Computer aided detection
CPM: Competition performance metric
CNN: Convolutional neural network
CT: Computed tomography
DCNN: Deep convolutional neural network
DNN: Deep neural network
FROC: Free response receiver operating characteristic
LIDC-IDRI: Lung image database consortium and image database resource initiative
LUNA16: LUng nodule analysis 2016
RF: Random forest
